# The application of the drug user quality of life scale (DUQOL) in Australia

**DOI:** 10.1186/1477-7525-10-31

**Published:** 2012-03-18

**Authors:** Carlos Zubaran, Jonathan Emerson, Rishi Sud, Elham Zolfaghari, Katia Foresti

**Affiliations:** 1 School of Medicine, University of Western Sydney, Department of Psychiatry, Western Sydney Local Health District, Blacktown Hospital, PO Box 6010, Blacktown, NSW 2148, Australia; 2 School of Psychology, University of Western Sydney, Department of Psychiatry, Western Sydney Local Health District, Blacktown Hospital, PO Box 6010, Blacktown, NSW 2148, Australia; 3 Blacktown Hospital, Department of Psychiatry, Western Sydney Local Health District, Blacktown Hospital, PO Box 6010, Blacktown, NSW 2148, Australia; 4 Department of Psychiatry, Western Sydney Local Health District, Blacktown Hospital, PO Box 6010, Blacktown, NSW 2148, Australia

**Keywords:** Quality of life, Questionnaires, Substance-related disorders, Validation studies, Australia

## Abstract

**Background:**

The concept of quality of life relates to the perceptions of individuals about their mental and physical health as well as non-health related areas. The evaluation of quality of life in the context of substance abuse has been conducted using generic instruments. The Drug Users Quality of Life Scale (DUQOL) is a specific assessment tool in which the most pertinent and salient areas to drug abusers are taken into consideration. In this study, the authors report the results of a validation study in which the DUQOL was used for the first time in Australia.

**Methods:**

A sample of 120 participants from inpatient and outpatient treatment facilities completed a series of questionnaires, including the DUQOL and the World Health Organization Quality of Life Assessment-BREF (WHOQOL-BREF). Parameters investigated in this study included the demographic characteristics of the sample, internal structure, and convergent validity. Correlations between the DUQOL scale scores and the scores of the WHOQOL-Bref test were investigated via Pearson product-moment correlation analyses.

**Results:**

The English version of the DUQOL attained a significant overall Cronbach's alpha of 0.868. The factorial analysis of the DUQOL identified one principal factor that accounted for 28.499% of the variance. Convergent validity analyses demonstrate significant correlations (*p *< 0.01) between the DUQOL scores and the scores of all four dimensions of the WHOQOL-BREF questionnaire.

**Conclusions:**

This study demonstrates that the DUQOL constitutes a reliable research instrument for evaluating quality of life of substance users in Australia.

## Introduction

The field of quality of life (QoL) measurement has been evolving as a formal discipline with structured theoretical foundations and a specific methodology for over 30 years [1]. Quality of life has become increasingly recognized as an important outcome measure in treatment studies and health service research [2]. The evaluation of QoL is also widely used in clinical trials and in observational studies of health and disease with the aim of evaluating interventions as well as adverse effects of treatment and the impact of the disease process itself [3].

According to the World Health Organization (WHO), health can be defined as a state of complete physical, mental and social well-being [4]. Yet, the conceptual boundaries of QoL has been re-examined in order to include additional elements such as sociocultural conditions, as well as factors contributing to mental and physical health, which ideally will transcend the rather circumscribed dichotomy of the health-disease process [5]. Gill and Feinstein [6] defined QoL as a reflection of respondents' perceptions and reactions to not only their mental and physical health, but also to non-health-related areas, including family, friends and work. A broader definition also includes life satisfaction, attainment in social and professional roles, a sense of being productive, a sense of control over one's destiny, as well as a pleasurable and satisfying sense of existence and spiritual fulfillment [7]. Other definitions give salience to factors related to patients' health status and overall functioning, including absence of symptoms, physical aptitude, emotional aspects, cognitive capacity and overall sense of life-satisfaction [8].

Quality of life instruments have been distinguished as generic or specific tools [9]. The generic instruments are so-called due to the lack of a specific correlation with any particular disease process. Generic QoL measures cover a broad spectrum of dimensions related to QoL, including physical aptitude, social relationships, mental well-being and an overall perception of physical and general QoL. These questionnaires were conceived for use both in samples from the general population and from specific groups of patients with the goal of evaluating various diseases and the impact of their treatment [10]. The specific tools, on the other hand, were developed to evaluate the difficulties presented in a specific group of patients or associated with a specific disorder.

Quality of life is also becoming an important clinical and research outcome within the drug and alcohol abuse context. The subjective aspect of QoL, especially in the field of mental health, has achieved importance in the measurement of therapeutic results, which facilitated a gradual shift in clinical focus from identifying a cure to enhancing QoL [11]. Taking into account the usual chronic nature of substance dependence, it is important to establish a model of longitudinal monitoring, which helps to improve communication between patients and healthcare professionals [12].

In the area of substance abuse, the concept of QoL has been applied to evaluate functioning, well-being and life satisfaction [13,14]. Ideally, the objective of evaluating QoL among drug-dependent individuals should be not only to evaluate patients with regard to the presence or absence of symptoms or adverse reaction to treatment, but also to focus on how drug-dependent individuals experience their daily lives [15,16]. In the area of substance use, though, the evaluation of QoL of substance users has been conducted mainly using generic QoL instruments [17]. Some studies have focused preferentially on health-related factors, which overshadows the complexities of drug dependence or personal factors that may hinder effective treatment [15]. A recent generation of assessment tools is currently focusing on areas specifically related to substance use. In contrast to general QoL measures, which are structured around domains of similar importance, QoL tools specifically designed for substance use measurement allow for a specific selection of life areas that are most salient for drug users (DUs)[18].

The Injection Drug User Quality of Life Scale (IDUQOL) is a specific QoL assessment tool designed in Canada, which evaluates health and non-health related aspects of the injecting drug users, with an emphasis on individual circumstances and environmental factors. Many of the areas included in the IDUQOL "are particularly relevant to the physical, social, psychological, occupational, and geographical reality" of substance users. [[19], pg.3]. The psychometric properties of a preliminary 17-item English version of the Injection Drug User Quality of Life Scale (IDUQOL) were assessed in a sample of 61 participants [20]. Later, a validation study of a 21-item English version revealed that the IDUQOL total score measures a construct consistent with quality of life [19]. The 21-item version was adapted to Spanish and its psychometric properties were evaluated in a sample of 100 participants in Spain [21]. Subsequently, a 22-item English version was developed to further "evaluate overall quality of life in DUs in a particular region"[22]. The 22-item version, denominated Drug User Quality of Life Scale (DUQOL), was adapted and translated to Spanish for the assessment of users of both injectable and non-injectable drugs in Spain [23]. The Spanish version was considered a valid measure of subjective QoL among drug users, which can be used to assess changes in QoL as a result of interventions such as harm reduction strategies, health care services, housing initiatives, and drug treatment programs [23]. The current study was developed with the aim of assessing the psychometrics properties of the English version of the DUQOL (22 items) as used for the first time in Australia. To the authors' knowledge, this is the first time that the 22-item English version of this instrument is tested in a sample of treatment-seeking individuals.

## Methods

### Sample

This study evaluated 120 adults recruited from inpatient and outpatient treatment facilities within the Western Sydney Local Health District catchment area, Australia, from April to October 2010. Research sites included Blacktown Hospital, Cumberland Hospital, Nepean Hospital, and the Mount Druitt Centre for Addiction Medicine, all of which are higher education training facilities within Western Sydney Local Health District. Potential participants presenting for treatment were randomly invited to respond to the questionnaires. The inclusion criteria comprised (a) being above the age of 18; (b) fulfillment of the DSM-IV (Diagnostic and Statistical Manual of Mental Disorders, 4th Edition) criteria for any disorder related to substance use and (c) the ability to understand the aim of the study as well as the content of the questions in both questionnaires, which entailed a satisfactory command of English. Exclusion criteria comprised of presentations exclusively due to alcohol abuse and/or involuntary admission for inpatient treatment.

### Informed consent

This study was granted approval by the Western Sydney Local Health District Human Research Ethics Committee. Prospective participants were provided with a written protocol pertaining to the study and a verbal explanation about the purpose of the study. They were also informed that participation was voluntary, confidential and anonymous. Volunteers were also informed that they could withdraw from this study at any time without any repercussion to their treatments. Research participants were then asked to sign an informed consent form prior to their inclusion in the study.

### Interview process

Five data collectors underwent a period of training and supervision by the principal investigator prior to administering questionnaires independently. The data collectors met regularly to address any queries and ensure each were following the same procedure. The study participants completed the questionnaires under minimal guidance from the trained examiners, who followed standardized instructional procedures. Interviews took place in a suitable room at one of the research sites mentioned above. Occasionally, specific questions not considered in the initial instruction procedures were answered on a one-to-one basis. Particular care was taken with non-native English speaking participants in order to ensure a satisfactory understanding.

### Instruments

#### The drug users quality of life scale (DUQOL)

The DUQOL assesses an individual's QoL and satisfaction in 22 life domains. It is constituted of 7-point Likert scales, which range from 1 (very unsatisfied) to 7 (very satisfied) resulting an average total score of QoL, an average total score for important areas and an average total score for areas that are not important. Volunteers are asked to classify each life area as "important" or "unimportant" to them. Each life domain is portrayed on a 5 by 5 inch card, with the name of the domain, a representative picture on the front of the card, and a description of the domain on the back of the card. The sum of the 22 scores generates a mean overall quality of life score, such that the higher the score, the better the quality of life. The mean overall QoL score is expressed quantitatively and without cut-off thresholds. In this study, the English version of the DUQOL was used [22].

#### The world health organization quality of life assessment-BREF (WHOQOL-BREF)

The World Health Organization Quality of Life Assessment-BREF (WHOQOL-BREF), is a questionnaire developed by the WHO as an abbreviated 26-item version of the WHOQOL-100 instrument for the assessment of quality of life across various cultural settings [24]. The WHOQOL-BREF is divided into four domains: Physical, Psychological, Social Relationships, and Environment. Each domain score reflects an individual's perception of his or her quality of life in that particular area. Two additional questions examine the individual's overall perception of (1) quality of life and (2) health status. The WHOQOL-BREF has been validated across a wide range of languages.

In addition to the DUQOL and WHOQOL-BREF, research participants were also asked to complete forms related to demographic information including age, sex, ethnicity, highest level of educational attainment, employment status, and most frequently consumed drugs.

### Databank configuration

One patient did not complete the DUQOL questionnaire due to emergence of symptoms of restlessness during the interview. Three volunteers did not complete a subset of questions related to "important and non-important" life areas. Two research participants did not complete the two final questions of the DUQOL questionnaire, in which cases the mean scores of the first 20 questions were used as a proxy value. All remaining DUQOL and WHOQOL-BREF questionnaires were completed properly and therefore given full statistical consideration.

### Statistical analysis

Descriptive statistics were used to analyze demographic information. The analysis of internal consistency of the English version of the DUQOL tested in Australia was determined by generating Cronbach's alpha indices for each question of this assessment tool. The factorial analysis of the same scale was conducted by Maximum Likelihood Factor analysis with Varimax rotation. Application of the scree test was performed to identify the most meaningful factorial structure.

A series of correlations between the DUQOL scale scores and the scores of each dimension of the WHOQOL-Bref test were investigated via Pearson product-moment correlation analyses. All analyses were conducted with IBM SPSS Statistics^® ^software.

## Results

### Demographic information

The sample consisted of 76 males (63.3%) and 44 females (36.7%). The mean age of the sample was 37.68 years (SD = 10.41), and there was no significant age difference between male and female participants. In terms of educational attainment, 66.1% had not completed secondary school, 18.2% had completed secondary school, 12.4% had completed vocational studies, and 3.3% had completed a university degree or higher. In terms of occupational status, 43% of the sample was employed at the time of the study, 38% were unemployed, 4.1% were studying, and 14.9% did not disclose their employment status.

### Internal structure information

#### Reliability data

The internal consistency (correlation of the items with the total score) of the English version of the DUQOL is shown in Table 1. All items included in the DUQOL attained significant Cronbach's alpha of 0.853 or superior. The DUQOL overall Cronbach's alpha totaled 0.868. Table 2 presents the inter-item correlation matrix for the DUQOL, with correlations between all areas and total score. The correlation scores ranged from 0.241 to 0.778 and the mean of the scores was 0.491.

**Table 1:** The English version of the Drug User Quality of Life Scale Scale Item Analysis and Reliability Data (*n *= 120)

		Response percentages									
					
Item		1	2	3	4	5	6	7	Mean	Standard Deviation	Cronbach's Alpha if Item Deleted
1.	Being useful	20	5	6.7	17.5	11.7	12.5	26.7	4.40	2.23	0.863
2.	Drugs	31.7	11.7	6.7	20	5	3.3	21.7	3.52	2.31	0.874
3.	Drug treatment	7.5	1.7	3.3	22.5	9.2	18.3	37.5	5.29	1.82	0.865
4.	Education	16.7	10.8	6.7	25	9.2	12.5	19.2	4.13	2.08	0.867
5.	Family	14.2	10	7.5	11.7	10	12.5	34.2	4.67	2.24	0.862
6.	Feeling Good	22.5	12.5	7.5	15	16.7	12.5	13.3	3.82	2.11	0.853
7.	Friends	16.7	5.8	14.2	17.5	11.7	12.5	21.7	4.26	2.10	0.861
8.	Harm reduction	0.8	10.8	3.3	0.8	25	5	9.2	5.15	2.10	0.867
9.	Health	20	12.5	7.5	17.5	11.7	15.8	15	3.96	2.12	0.858
10.	Health care	5	6.7	6.7	15	8.3	16.7	41.7	5.32	1.89	0.863
11.	Housing	18.3	2.5	3.3	9.2	7.5	17.5	41.7	5.04	2.29	0.863
12.	Free choice	5	7.5	5.8	20.8	11.7	18.3	30.8	5.05	1.83	0.858
13.	Leisure activities	20.8	10.8	10.8	13.3	12.5	15	16.7	3.98	2.16	0.859
14.	Money	29.2	13.3	12.5	17.5	7.5	8.3	11.7	3.33	2.08	0.864
15.	Neighborhood safety	14.2	7.5	3.3	15	10.8	19.2	30	4.78	2.15	0.864
16.	Partner(s)	20	6.7	3.3	37.5	2.5	6.7	23.3	4.09	2.12	0.865
17.	Community resources	5	5	3.3	25	17.5	23.3	20.8	4.98	1.65	0.863
18.	Sex	25.8	5.8	0.8	33.3	5.8	8.3	20	3.93	2.19	0.866
19.	Spirituality	12.5	2.5	1.7	29.2	10	11.7	32.5	4.87	2.00	0.865
20.	Transportation	12.5	7.5	8.3	15.8	10.8	17.5	27.5	4.67	2.09	0.866
21.	Sense of future	12.5	7.5	12.5	19.2	11.7	15	21.7	4.42	2.02	0.857
22.	How other treat you	8.3	9.2	8.3	20	7.5	27.5	19.2	4.68	1.91	0.856

*Rating scale: 1 = Very dissatisfied; 2 = Moderately dissatisfied; 3 = Slightly dissatisfied; 4 = neutral (neither dissatisfied nor satisfied); 5 = slightly satisfied, 6 = moderately satisfied, and 7 = very satisfied.

**Table 2:** The inter-item correlation matrix for the DUQOL, with correlations between all areas and total score

	1	2	3	4	5	6	7	8	9	10	11	12	13	14	15	16	17	18	19	20	21	22
**1**	1																					
**2**	0.04	1																				
**3**	0.076	0.104	1																			
**4**	0.195*	0.069	0.144	1																		
**5**	0.195*	0.150	0.284 **	0.136	1																	
**6**	0.537**	0.193*	0.301 **	0.248 **	0.479 **	1																
**7**	0.308**	0.097	0.169	0.128	0.265 **	0.427**	1															
**8**	0.123	0.179*	0.244**	0.093	0.203*	0.216*	0.096	1														
**9**	0.396**	0.159	0.205*	0.167	0.301 **	0.732**	0.244**	0.206*	1													
**10**	0.205*	0.060	0.297**	0.241**	0.151	0.313**	0.193*	0.203*	0.348**	1												
**11**	0.177	-0.041	0.121	0.226*	0.340**	0.279**	0.205*	0.113	0.343**	0.210*	1											
**12**	0.244**	0.133	0.239**	0.302**	0.287**	0.499**	0.272**	0.279**	0.402**	0.218*	0.303**	1										
**13**	0.400**	0.055	0.117	0.184*	0.288**	0.519**	0.392**	-0.007	0.294**	0.164	0.257**	0.424**	1									
**14**	0.289**	-0.044	0.192*	0.153	0.014	0.333**	0.175	0.135	0.245**	0.132	0.270**	0.349**	0.396**	1								
**15**	0.159	0.123	0.123	0.112	0.151	0.311**	0.342**	0.130	0.313**	0.164	0.418**	0.257**	0.196*	0.221*	1							
**16**	0.083	006	0.123	0.090	0.386**	0.265**	0.373**	0.265**	0.235**	0.141	0.244**	0.294**	0225*	0.094	0.100	1						
**17**	0.157	0.095	0.292**	0.211*	0.124	0.291**	0.219*	0.194*	0.201	0.478**	0.238**	0.334**	0.254**	0.202*	0.296**	0.041	1					
**18**	0.189*	0.059	0.077	0.008	0.300**	0.258**	0.436**	0.192*	0.173	0.154	0.024	0.179	0.339**	0.173	0.059	0.480**	-0.024	1				
**19**	0.170	0.137	0.149	0.242**	0.189	0.282**	0.162	0.094	0.232*	0.317**	0.195*	0.276**	0.338**	0.234**	0.202*	0036.	0.187*	0.103	1			
**20**	0.228*	-0.137	0.269**	0.247**	0.099	0.176	0.220*	0.059	0.092	0.169	0.329**	0.351**	0.240**	0.326**	0.332**	0.088	0.332**	0.103	0.032	1		
**21**	0.398**	0.067	0.352**	0.267**	0.330**	0.583**	0.258**	0.252**	0.449**	0.286**	0.297**	0.326**	0.466**	0.410**	0.114	0.234*	0.275**	0.260**	0423**	0.209*	1	
**22**	0.262**	0.277**	0.366**	0.129	0.392**	0.594**	0.478**	0.337**	0.485**	0.353**	0.353**	0.405**	0.321**	0.182*	0.441**	0.340**	0.336**	0.281**	0261**	0.199*	0.413**	1
**T**	0.515**	0.241**	0.446**	0.400**	0.540**	0.778**	0.570**	0.402**	0.636**	0.495**	0.524**	0.640**	0.480**	0.490**	0.451**	0.483**	0.432**	0.458**	0.430**	0.671**	0.716**	

* Correlation is significant at 0.05 level (2-tailed)

** Correlation is significant at 0.01 level (2-tailed)

Items: 1 = Health; 2 = Drugs and Alcohol; 3 = Drug and Alcohol Treatment; 4 = Education and Training; 5 = Family; 6 = Feeling Good About Yourself; 7 = Friends; 8 = Harm Reduction; 9 = Being Useful; 10 = Health Care; 11 = Housing;

12 = How Others Treat You; 13 = Free Choice; 14 = Leisure Activities; 15 = Money; 16 = Neighborhood safety; 17 = Partner(s); 18 = Community resources; 19 = Sex; 20 = Sense of future; 21 = Spirituality; 22 = Transportation; T = Total Score.

#### Factorial analysis

**Figure 1:**
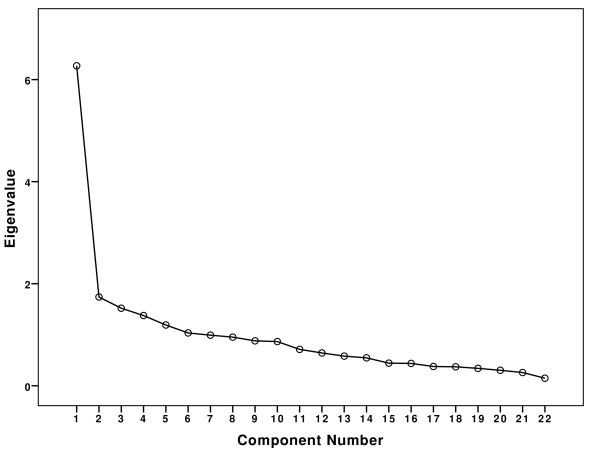
**Scree plot of the eigenvalues**.

Maximum likelihood factor analysis with Varimax rotation was conducted on the ratings given by the 120 respondents to the 22-item DUQOL. An initial exploratory factor analysis yielded six factors with eigenvalues exceeding unity, which accounted for 59.693% of the variance. As demonstrated in Figure 1, application of the scree test suggested that a single factor seemed warranted. The one factor solution appeared the most tenable option, given that factor one contributed with 28.499% of the variance.

### Convergent validity

The results of correlation analyses revealed statistically significant correlation coefficients between the average total DUQOL quality of life score and the scores of different dimensions of the WHOQOL-Bref questionnaire. The magnitude of the correlations are as follows: r = 0.56, *p *< 0.01 (DUQOL × Physical Domain - D1); r = 0.59, *p *< 0.01 (DUQOL × Psychological Domain - D2); r = 0.62, *p *< 0.01 (DUQOL × Social Domain - D3); and r = 0.69; *p *< 0.01 (DUQOL × Environmental Domain - D4).

### Substance use

The sample used an average of 2.1 different drugs (SD = 1.1). The most frequently used drugs were cannabis (55.4%), opioids (45.5%), and alcohol (30.6%). Other common drugs of choice were methamphetamine ("speed") (28.1%), cocaine (11.6%), benzodiazepines (used illicitly) (10.6%), ecstasy (8.3%), and other amphetamines (6.6%).

### Quality of life - average total score

**Figure 2:**
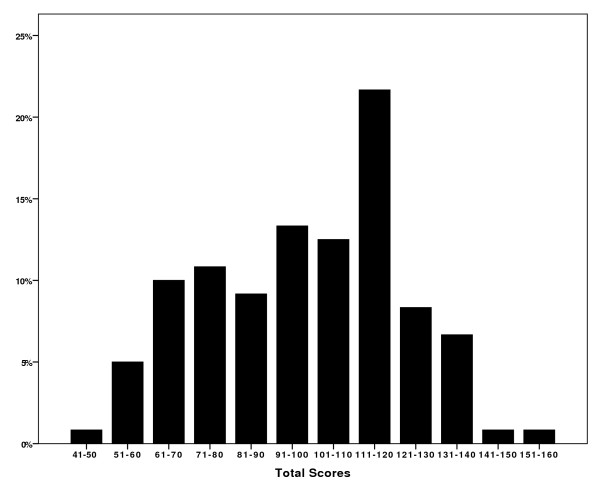
**The distribution of the DUQOL total scores in the sample (n = 120)**.

The mean overall score according to the DUQOL scale in this sample (*n *= 120) was 98.3 (SD = 23.5). The scores ranged from 49 to 151, which indicate that the application of the DUQOL scale to this population group achieved neither a ground nor a ceiling effect. Figure 2 shows the grouped distribution of the average total quality of life scores.

### Quality of life - overall self-rating

**Figure 3:**
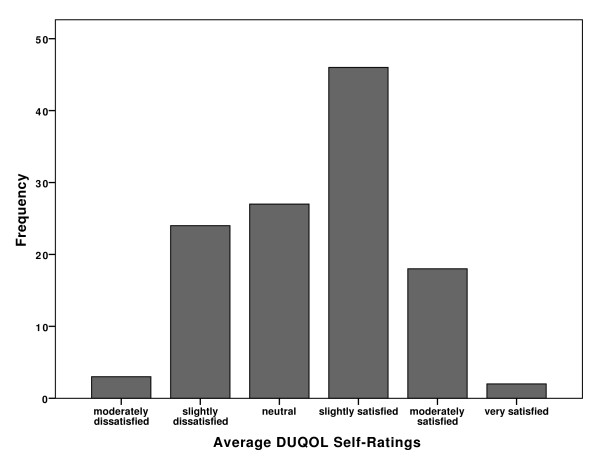
**The percentage of participants according to the overall level of satisfaction with their quality of life**.

The overall self-rating of quality of life varied from "very satisfied" (1.7% of the sample) to "slightly satisfied" (38.3%). Mid-range percentages were as follows: "moderately dissatisfied" (2.5%); "slightly dissatisfied" (20%); "neutral" (22.5%); and "moderately satisfied" (15%). Figure 3 presents graphically the results mentioned above.

### Quality of life - salience of specific life areas

**Figure 4:**
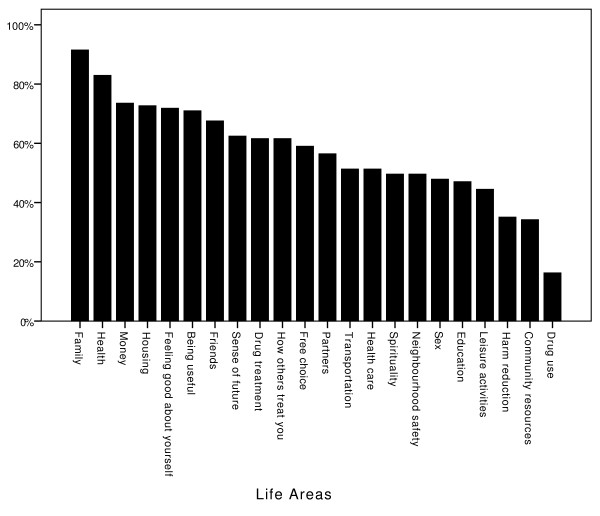
**The percentage of participants who regarded each specific life area as important to their quality of life**.

The two areas most frequently considered "important" for participants' quality of life was family (91.5%) and health (82.9%). The areas least frequently considered important were drug use (16.2%), community resources (34.2%), and harm reduction (35.0%). Figure 4 shows the percentage of participants who regarded each life area as important to their quality of life.

### Ease of completion

The majority of research participants found it easy to complete the DUQOL scale. In this sample, 55.8% of the sample rated the completion process as "Not difficult at all", 25% found it "A little difficult", 14.4% found it "Moderately difficult", and 4.8% reported it as "Extremely difficult". The mean time taken to complete the DUQOL scale was 9.24 (SD = 3.88) minutes.

## Discussion

Overall, the results of this study provide evidence for the reliability, validity and usefulness of the Drug User Quality of Life Scale (DUQOL) as a practical instrument for assessing quality of life in drug users. In this context, reliability refers to the consistency of a set of measurements or a measuring instrument, whereas validity refers to he extent to which a measurement is well-founded and corresponds accurately to the real world, measuring what it claims to measure [25]. This is especially relevant given that the availability of research tools specifically designed to assess quality of life among substance users is currently limited [18]. The methodology executed in this study followed the approach used in additional validation studies of other psychometric tools conducted by the same research group [26,27].

In regard to demographic information, approximately two thirds of the sample comprised of males (63.3%) and the gender distribution observed in this study, on the whole, matches the prevalence rates of substance use by gender in Australia [28]. A prototypical description of this sample would be a man in his late 30s who has completed secondary school, possibly employed and seeking treatment. There has been well-documented evidence that "cannabis remains the most commonly used illicit drug in Australia" [28] and that "a need for increased treatment provision is likely to be largely driven by an ageing cohort of cannabis users, with daily smoking of cannabis now most commonly reported among 30-39 year olds" [28]. In addition, the literature indicates that the majority of those who seek treatment for problems related to cannabis as their principal drug of concern had difficulties with another substance [29]. Therefore, in light of the foregoing, the demographic data and drug use findings obtained in the current study are consistent with previous epidemiological evidence collected in Australia.

The English version of the DUQOL proved to have sound psychometric properties. Internal consistency data revealed significant Cronbach's alpha coefficients for all 22 areas of the DUQOL. The internal consistency of the English version of the DUQOL demonstrates that the instrument coherently investigates the construct of quality of life as measured uniformly by its items. The reliability analysis of data generated in this research was conducted according to parameters described in similar studies developed by other investigators [26,27].

The results of the DUQOL factorial analysis demonstrated that the English version of the DUQOL tested in this study is structurally unidimensional. Although six factors were extracted in an exploratory factor analysis, the scree test revealed one main factor. Different criteria have been proposed for establishing the number of factors to extract based on the magnitudes of the eigenvalues. On criterion is to retain all factors that have eigenvalues greater than the unity, whereas another method is to examine the scree plot and to retain factors with eigenvalues in the sharp descent part of the plot before the eigenvalues start to plateau [30]. A factorial analysis based on the results of the scree test yields accurate results more often than the extraction method based on eigenvalues with values grated than the unity [30].

In addition, a significant intercorrelation among all items in the DUQOL as demonstrated in Table 2 confirms further the sound internal consistency of this instrument. It has been advocated that "one can achieve a high internal consistency reliability estimate by having either many items or highly intercorrelated items (or some combination of the two)"[31]. In fact, the average inter-item correlation has been considered "much more useful index than coefficient alpha per se". The mean inter-item correlation score of the DUQOL was 0.491, which falls within the recommended range of 0.15-0.50 [32].

An interesting highlight of the current study was that the mean DUQOL scale score was similar to findings observed in a study conducted in Spain wherein the Spanish version of the DUQOL was validated [23]. In the latter study, the correlations between areas and the total ranged from 0.34 to 0.64. The validation study conducted in Spain established the criterion validity of the Spanish version by demonstrating significant correlations, in the expected directions, with a series of dichotomous variables. However, a potential weakness of this investigation was that the Spanish version of the DUQOL was not compared to another QoL assessment tool. In contrast, convergent validity in the current study was established by comparing the DUQOL with the four domains of the WHOQOL-Bref questionnaire. There were significant correlations between the DUQOL scores and the scores of the four domains of the WHOQOL-Brief questionnaire, thereby, suggesting confirmatory evidence that the DUQOL validly assesses the quality of life of substance users in this sample.

Other findings related to the DUQOL indicated that this instrument was easy to administer, well accepted by most of the research participants and that the majority of the sample found it easy to complete the questionnaire. The latter claim is further substantiated by the reduced mean amount of time needed to complete the test. As observed elsewhere, questionnaires that investigate health status and quality of life in multiple domains and with a larger number of questions tend to take longer to complete [33,34]. Methodologically, the acceptability of an instrument within a target population is an important element to take into account when considering the widespread use of a tool. This is especially important as many surveys use QoL assessment tools in conjunction with other assessment tools and forms.

Given that validation studies can potentially be more prone to methodological limitations when conducted over the telephone [35], a distinct advantage of the current study was the use of face-to-face interview technique. An additional strength of the current study can be seen in the recruitment setting. The study was conducted entirely within the public health care system, which allowed for the inclusion of all prospective research participants, irrespective of private health care insurance cover or employment status. Therefore, the results presented here provide a representative analysis of the quality of life of substance users in urban areas of Australia. Conversely, considering that environmental factors are known to influence substance use and mental health in Australia, via circumstances like droughts, fires, and limited access to health care facilities due to remoteness and isolation [36], the fact that samples were recruited exclusively from urban health care facilities may be considered as possible limitation in the present study.

## Conclusion

This study demonstrates that the DUQOL and the WHOQOL-Bref, which are conceptually designed tools to assess quality of life, present significant constructural correlations and are therefore congruent. The internal consistency of the DUQOL was satisfactory, which demonstrates that the instrument coherently investigates the quality of life as measured uniformly by its items. The DUQOL was well accepted by all volunteers. They answered the questions without experiencing significant difficulties, which demonstrates the ease of use of the instrument. The results presented here indicate that the DUQOL scale represents a reliable and valid measurement tool for the assessment of QoL among substance users in Australia.

## Abbreviations

DUQOL: Drug users quality of life scale; IDUQOL: Injecting drug users quality of life scale; QoL: Quality of life; WHO: World Health Organization; WHOQOL-BREF: World Health Organization Quality of Life Assessment-BREF; DSM IV: Diagnostic and statistical manual of mental disorders, 4th edition.

## Competing interests

The authors declare that they have no competing interests.

## Authors' contributions

CZ participated in the conception of study design and research project; application to the ethics and research committees, data collection, statistical analysis of data, interpretation of findings, writing of the manuscript and its submission to scientific journal. JE and RS participated in the data collection, statistical analysis of data, interpretation of findings, writing of the manuscript. EZ data collection, statistical analysis of data, interpretation of findings, writing of the manuscript and its submission to scientific journal. KF participated in the application to the ethics and research committees, data collection, statistical analysis of data, writing of the manuscript and its submission to scientific journal. All authors read and approved the final manuscript.
